# Musculoskeletal disorders among construction workers: a one-year follow-up study

**DOI:** 10.1186/1471-2474-13-196

**Published:** 2012-10-13

**Authors:** Julitta S Boschman, Henk F van der Molen, Judith K Sluiter, Monique HW Frings-Dresen

**Affiliations:** 1 Academic Medical Center, Coronel Institute of Occupational Health, University of Amsterdam, Amsterdam, the Netherlands; 2 Arbouw, Dutch Health & Safety Institute in the Construction Industry, Harderwijk, The Netherlands

**Keywords:** Construction industry, Longitudinal study, Work-related musculoskeletal disorders

## Abstract

**Background:**

Work-related musculoskeletal disorders (MSDs) are an important cause of functional impairments and disability among construction workers. An improved understanding of MSDs in different construction occupations is likely to be of value for selecting preventive measures. This study aimed to survey the prevalence of symptoms of MSDs, the work-relatedness of the symptoms and the problems experienced during work among two construction occupations: bricklayers and supervisors.

**Methods:**

We randomly selected 750 bricklayers and 750 supervisors resident in the Netherlands in December 2009. This sample was surveyed by means of a baseline questionnaire and a follow-up questionnaire one year later. The participants were asked about complaints of the musculoskeletal system during the last six months, the perceived work-relatedness of the symptoms, the problems that occurred during work and the occupational tasks that were perceived as causes or aggravating factors of the MSD.

**Results:**

Baseline response rate was 37%, follow-up response was 80%. The prevalence of MSDs among 267 bricklayers and 232 supervisors was 67% and 57%, respectively. Complaints of the back, knee and shoulder/upper arm were the most prevalent among both occupations. Irrespective of the body region, most of the bricklayers and supervisors reported that their complaints were work-related. Complaints of the back and elbow were the most often reported among the bricklayers during work, whereas lower arm/wrist and upper leg complaints were the most often reported among the supervisors. In both occupations, a majority of the participants perceived several occupational physical tasks and activities as causes or aggravating factors for their MSD. Recurrent complaints at follow-up were reported by both bricklayers (47% of the complaints) and supervisors (31% of the complaints). Participants in both occupations report that mainly back and knee complaints result in additional problems during work, at the time of follow-up.

**Conclusions:**

A substantial number of the bricklayers and the supervisors report musculoskeletal disorders, mainly back, knee and shoulder/upper arm complaints. The majority of the bricklayers and half of the supervisors believe that their complaints are work-related. Irrespective of occupation, participants with MSDs report substantial problems during work. Workplace intervention measures aimed at occupational physical tasks and activities seem justified for both occupations.

## Background

The construction industry is known for its occupational risks and hazards and the associated adverse health effects [1-3]. For construction workers, musculoskeletal disorders (MSDs) are a main cause of productivity loss at work [4], functional impairments [5] and permanent disability [6]. However, workers in different construction occupations are at risk for different work-related MSDs [7]. This is mainly due to different biomechanical risk factors [7-9].

Over the years, primary prevention has improved and the biomechanical load in several physically demanding construction occupations has been reduced [10-15]. Primary prevention is seen as the key to eliminating work demands that are too high for the workers and reducing the risk of adverse health effects. However, results from long-term follow-up studies do not show a significant preventive effect for MSDs [11,12]. Therefore, MSDs among construction workers continue to be a problem. Recent results from Oude Hengel et al. [16] showed that, in a population of currently working construction workers, more than half suffered from occasional or frequent musculoskeletal complaints. These complaints reduce the workers’ ability and willingness to continue to remain in their job until retirement [16]. To retain workers in the construction field, it is essential to monitor these complaints, select potentially effective intervention measures and prevent these workers from further physical deterioration.

Preventive measures or intervention programs should be tailored to the individual needs of workers with MSDs [17,18]. For example, each individual’s personal characteristics, behaviour, occupational demands and possible work modifications should be assessed [19]. However, based on the high physical demands placed on construction workers and their risks for MSDs, it seems legitimate to focus on the workplace first. Because of the wide variety of construction occupations, we have chosen to focus on two distinct occupations in the present study: bricklayers and supervisors. Based on previous research, we know that both occupations are at risk for musculoskeletal complaints, although their physical demands differ remarkably [7]. Bricklayers, for example, are known to have an increased risk for complaints related to the musculoskeletal system, particularly lower back pain [20,21]. Based on the physically demanding nature of the job, this is hardly surprising. However, there is also evidence suggesting an increased risk for complaints of the musculoskeletal system among individuals with mentally demanding positions in the construction industry, such as that of the construction supervisor [8,22].

Knowledge of the risk factors for MSDs is the first step in an evidence-based workplace-oriented approach to addressing these problems. Based on the literature, lower back complaints among bricklayers might be related to lifting and carrying [23], kneeling [24] or prolonged standing [25]. Complaints of the neck and upper extremities among the supervisors might be symptoms of stress [26,27] or they might be caused by the prolonged use of a computer in a static, sitting position [28]. These examples illustrate that there might be multiple risk factors for specific MSDs in specific occupations. Knowledge of the risk factors will therefore be helpful, but our understanding is not yet sufficient to guide the selection of the most effective intervention measures and eliminating all risk factors at once is not feasible. It seems appropriate to first select those preventive measures associated with the tasks or activities that either cause or aggravate the complaint [29] or lead to restrictions in work functioning.

An improvement in our understanding of MSDs at the population level will facilitate the implementation of preventive measures and individually tailoring workplace programs. Therefore, in the present study, we aim to provide a more in-depth view of MSDs among two construction occupations. The main research question in this questionnaire survey with a follow-up period of one year is:

1. What is the six-month prevalence of self-reported long-lasting or regular musculoskeletal complaints among bricklayers and supervisors and to what extent are these complaints recurrent over the course of one year?

Among the bricklayers and supervisors reporting long-lasting or regular musculoskeletal complaints we asked:

2a. To what extent are these musculoskeletal complaints perceived as work-related by the worker?

2b. What is the extent of the experienced problems during work, and does this change over the course of one year?

2c. What occupational tasks or activities were perceived as causes or aggravating factors for the complaints?

## Methods

### Sample and procedure

A priori we aimed at estimating the prevalence of MSDs with a precision of 5-6%, and expected that the prevalence of MSDs would be around 40% [8]. This leads to an estimated sample size of 257–369 among both occupations. Therefore, we aimed at including 300 participants for each occupation. Based on previous results we expected the response rate to be around 40% [30]. Therefore, we randomly selected 750 bricklayers and 750 construction supervisors from a Dutch registry comprised of all employed Dutch construction workers. The random selection was performed by the independent data manager of the registry, frequently assisting in selecting samples for research purposes.

Among the bricklayers were both those working in the construction of new buildings as in renovation. Among the construction supervisors were those working in ground, road and water construction and in commercial and industrial building. Furthermore, the selection was not restricted based on the type of construction supervisor (main or assisting supervisor).

In the collective labour agreement of the workers is recorded that research is performed under the authority of Arbouw, The Health and Safety Institute for the Dutch Construction Industry. The workers are reminded in writing of this agreement and informed about the aims of the present research. Workers can choose whether or not to participate voluntarily. The workers are aware of the fact that by filling in and returning the questionnaire, they give the researchers written consent to use their information anonymously for research purposes. The survey was conducted from December 2009 to January 2011 and consisted of a baseline questionnaire and a follow-up questionnaire one year later. At baseline, all participants received a sealed envelope at their home address containing a postcard with an invitation to participate, a questionnaire survey and an incentive (lottery ticket for a national lottery) [30]. At follow-up, only those who had responded at baseline were sent a second postcard, a follow-up questionnaire and an incentive (lottery ticket). Completing the questionnaires took approximately 20 min. The participants were asked to fully complete and return the questionnaire within two weeks. One reminder containing a postcard was sent to all participants after one week.

### Questionnaire

The questionnaire comprised multiple parts involving health-related topics, but to address the present research questions, only the items regarding musculoskeletal complaints and the associated or aggravating tasks and activities, are reported.

#### Work-related musculoskeletal complaints

We used an adapted version of a questionnaire on MSDs, which has been in previous research [11,12] and among other professions [31]. For every region of the body (shoulder and upper arm, elbow, lower arm and wrist, hand, hip, upper leg, knee, lower leg and ankle, foot, neck, back), participants were asked to indicate whether they have had regular or long-lasting complaints during the last six months (yes/no). If so, they were asked whether the complaints were caused by their work (yes/no) and the degree to which they experienced problems during their work (0 = no problems, 10 = many problems).

#### Tasks and activities

For every MSD, the participant was asked to indicate (yes/no) which tasks or activities caused or aggravated their musculoskeletal complaint. Based on a previous systematic literature review [7], the following tasks and activities were included: standing, kneeling and stooping, working with a bent back, carrying and lifting, repetitive arm-hand movements, working above shoulder height, working with vibrating hand tools, climbing a ladder/scaffold and driving vehicles. The construction supervisors were also asked about the activity ‘walking across the construction site’. The bricklayers were not asked about the activity walking, because this was considered not to be a main activity of the bricklayer. Additionally, the participants were asked to describe the cause or aggravator of their complaints if it was not represented in the list.

### Analysis

The analyses were performed separately for the two occupational groups. Information from the open-ended question on perceived causes or aggravating factors was categorised and described qualitatively. Descriptive statistics were presented as the percent, median and Inter Quartile Range (IQR). To gain insight in the characteristics and representativeness of the respondents, we tested i) for differences in age between responders and the initial sample using a t- test and ii) the relation between age (years) and having MSDs at baseline, and the relation between having or having not MSDs at baseline and response at follow-up using univariate logistic regression analysis. Statistical significance was set to an alpha level of 0.05. The prevalence and 95% confidence interval (CI) of the MSDs was calculated using the baseline data. Confidence intervals were calculated by using the Wald method when the sample consisted of more than 150 persons and the adjusted Wald method when the sample consisted of less than 150 persons [32].

Problems experienced during work was recoded into three categories (0–3 = no/little, 4-6= medium, 7–10 = many problems). Changes in the problems experienced during work were assessed by calculating the difference in the score between baseline and follow-up. We defined a difference over time in problems during work as a decrease or increase of 2 points or more. Furthermore, we assessed for every body region the frequencies and percentages of the reported tasks or activities that caused or aggravated the complaints of the body region under consideration. Next, for every task or activity a top-three of involved body regions was constructed and reported. The IBM SPSS Statistics 19 software was used to analyse the data.

## Results

### Characteristics of the study population

At baseline, the overall response rate was 39% for the bricklayers (292/750) and 34% for the construction supervisors (256/750). After excluding partially completed questionnaires, response rate was 36% (n=267) for the bricklayers and 31% (n=232) for the supervisors. No female workers responded. At follow-up, the overall response rate was 80%; 83% for the bricklayers (222/267) and 76% for the supervisors (177/232). The characteristics of the participants who responded at both baseline and follow-up, are shown in Table 1.

**Table 1:** Demographic and job characteristics among bricklayers and construction supervisors participating in both a baseline and a one-year follow up survey, December 2009 to January 2011

	**Bricklayers (n=222)**		**Construction supervisors (n=177)**	
	**Median**	**IQR**	**Median**	**IQR**
Age (years)	50	13	51	14
Years employed in construction	32	16	33	17
Years employed in present occupation	27	20	15	15
Years employed at present company	10	19	18	18
Working hours per week	40	0	45	10

*IQR* Inter Quartile Range.

We found no difference in age between the responding bricklayers and the initial sample of bricklayers. However, we did find a statistically significant difference in age for the construction supervisors. At baseline, the responding construction supervisors were older (5.5 years, p<0.01) than the sample.

Next to this, we found a statistically significant association between age (years) and response at follow-up for both bricklayers (OR 1.04 per year, 95% CI: 1.02-1.06) and construction supervisors (OR 1.03 per year, 95% CI: 1.00-1.05). Furthermore, we found a statistically significant association between age and having MSDs at baseline for the bricklayers (OR 1.02 per year, 95% CI: 1.00-1.04), but not the supervisors (OR 1.02 per year, 95% CI: 1.0-1.05. However, there was no relation between having versus not having MSDs at baseline and response at follow-up for both bricklayers (OR 1.09, 95% CI: 0.61-1.97) or supervisors (OR 0.85, 95% CI: 0.48-1.49).

### Prevalence of self-reported musculoskeletal health complaints

At baseline, two thirds of the bricklayers (67%, 179/267) reported one or more regular or long-lasting musculoskeletal complaint in the previous six months. The three body regions with highest prevalence were the back, knee and shoulder/upper arm (Table 2). Among the 179 bricklayers who reported complaints, most reported one complaint (37%, 66/179). However, approximately one third of the bricklayers reported two complaints (28%, 51/179), and among the bricklayers who reported complaints, 35% (62/179) reported three or more complaints.

**Table 2:** Prevalence (baseline) and recurrence (follow-up) of musculoskeletal complaints among Dutch bricklayers and construction supervisors participating in a baseline and Follow-up survey, December 2009 to January 2011

	**Bricklayers**						**Construction supervisors**					
	**Prevalence (MSD reported at baseline)**			**Recurrent complaints (MSD reported again at follow-up)**			**Prevalence (MSD reported at baseline)**			**Recurrent complaints (MSD reported again at follow-up)**		
**Body region**	**Relative frequency**	**%**	**(95% CI)**	**Relative frequency**	**%**	**(95% CI)**	**Relative frequency**	**%**	**(95% CI)**	**Relative frequency**	**%**	**(95% CI)**
Shoulder and upper arm	71/290	**24**	(19.5-29.4)	38/71	**54**	(42.0-64.6)	43/246	**17**	(12.7-22.2)	16/43	**37**	(24.3-52.1)
Elbow	36/285	**13**	(8.8-16.5)	10/36	**28**	(15.7-44.1)	16/246	**7**	(3.4-9.6)	3/16	**19**	(5.8-43.8)
Lower arm and wrist	40/281	**14**	(10.1-18.3)	14/40	**35**	(22.1-50.6)	9/247	**4**	(1.3-6.0)	2/9	**22**	(5.3-55.7)
Hand	25/283	**9**	(5.5-12.1)	9/25	**36**	(20.2-55.6)	19/249	**8**	(4.3-10.9)	8/19	**42**	(23.1-63.8)
Hip	32/284	**11**	(7.6-14.9)	11/32	**34**	(20.3-51.8)	23/249	**9**	(5.6-12.8)	9/23	**39**	(22.1-59.2)
Upper leg	14/284	**5**	(2.4-7.4)	8/14	**57**	(32.6-78.7)	14/245	**6**	(2.8-8.6)	4/14	**29**	(11.3-55.0)
Knee	75/285	**27**	(21.4-31.8)	42/75	**56**	(44.7-66.7)	37/249	**15**	(10.4-19.3)	15/37	**41**	(26.3-56.5)
Lower leg and ankle	20/282	**7**	(4.1-10.1)	7/20	**35**	(18.0-56.8)	20/247	**8**	(4.7-11.5)	4/20	**20**	(7.5-42.2)
Foot	23/284	**8**	(4.9-11.3)	11/23	**48**	(29.2-67.0)	26/248	**10**	(6.7-14.3)	0/26	**0**	(0–11.2)
Neck	36/284	**13**	(8.8-16.5)	18/36	**50**	(34.5-65.5)	38/248	**15**	(10.8-19.8)	14/38	**37**	(23.3-52.8)
Back	120/289	**42**	(35.8-47.2)	64/120	**53**	(44.4-62.0)	73/247	**30**	(23.9-35.2)	24/73	**33**	(23.2-44.3)

Among the supervisors, more than half of the respondents reported one or more regular or long-lasting musculoskeletal complaints (57%, 132/232). The highest prevalence of complaints was found for the back, the shoulder/upper arm, neck and knee (Table 2). Half of the supervisors with complaints, reported one complaint (68/132). Nearly one quarter (30/132) of the supervisors reported two complaints and another quarter of the respondents with complaints (34/132) reported three or more complaints.

### Recurrent complaints, work-relatedness and problems during work

#### Baseline

Overall, 81% (398/492) of the total number of MSDs experienced by the bricklayers were perceived as work-related. In particular, complaints of the elbow (100%, 36/36), back (90%, 108/120), neck (89%, 32/36) and lower arm and wrist (88%, 35/40) were typical complaints reported by the bricklayers as being work-related (Table 3). For the construction supervisors, 48% (154/318) of the reported MSD were described as work-related, and the complaints most often mentioned were associated with the hip (61%, 41/23), upper leg (57%, 8/14), lower arm/wrist (56%, 5/9) and back (53%, 39/73).

**Table 3:** Relative frequency and percentage of bricklayers and supervisors participating in a baseline and follow-up survey: i) perceiving their musculoskeletal complaints were work-related and ii) reporting they had many problems (≥7, on a scale from 0 to 10) during work due to their musculoskeletal complaints

**Body region**	**Bricklayers**						**Construction supervisors**					
	**MSD reported to be work-related**			**Having many problems during work**			**MSD reported to be work-related**			**Having many problems during work**		
	**Relative frequency**	**%**	**95% CI**	**Relative frequency**	**%**	**95% CI**	**Relative frequency**	**%**	**95% CI**	**Relative frequency**	**%**	**95% CI**
Shoulder and upper arm	61/71	**86**	(75.8-92.4)	29/70	**41**	(30.6-53.1)	21/43	**49**	(45.0-76.1)	16/42	**38**	(25.0-53.2)
Elbow	36/36	**100**	(91.7-100)	18/35	**51**	(35.6-67.0)	6/16	**38**	(18.4-61.5)	6/16	**38**	(18.4-61.5)
Lower arm and wrist	35/40	**88**	(73.4-95.0)	14/39	**36**	(22.7-51.6)	5/9	**56**	(26.6-81.2)	5/9	**56**	(26.6-81.2)
Hand	19/25	**76**	(56.3-88.8)	9/25	**36**	(20.1-55.6)	7/19	**37**	(19.1-59.1)	5/18	**28**	(12.2-51.2)
Hip	25/32	**78**	(61.0-89.3)	13/30	**43**	(27.4-60.8)	14/23	**61**	(40.7-77.9)	9/21	**43**	(24.4-63.5)
Upper leg	9/14	**64**	(38.6-83.8)	5/12	**42**	(19.3-68.1)	8/14	**57**	(32.6-78.7)	7/13	**54**	(29.1-76.8)
Knee	53/75	**71**	(59.5-79.8)	22/72	**31**	(21.1-42.0)	19/37	**51**	(35.9-66.6)	16/35	**46**	(30.5-61.8)
Lower leg and ankle	10/20	**50**	(29.9-70.1)	10/17	**59**	(36.0-78.4)	8/20	**40**	(21.8-61.4)	9/18	**50**	(29.0-71.0)
Foot	10/23	**43**	(25.6-63.2)	7/21	**33**	(17.1-54.8)	11/26	**42**	(25.5-61.1)	7/21	**33**	(17.1-54.8)
Neck	32/36	**89**	(74.1-96.2)	15/35	**43**	(28.0-59.2)	16/38	**42**	(27.8-57.8)	10/34	**29**	(16.7-46.3)
Back	108/120	**90**	(83.2-94.3)	54/111	**49**	(39.6-57.8)	39/73	**53**	(42.1-64.4)	23/68	**34**	(23.7-45.7)

Workers in both occupations were asked to indicate the extent of the problems that they experience as a result of their musculoskeletal complaints while doing their job. Most scores varied between five and seven for both occupations. About half of the bricklayers reported that they experience many problems during work (≥ 7) related to complaints of their lower leg/ankle, elbow and back (Table 3). About half of the participating supervisors reported that they experience many problems due to complaints of their lower arm/wrist, upper leg and lower leg.

#### Follow-up

At follow-up, there was a high percentage of recurrent MSDs among the bricklayers and the supervisors (Table 2). Among the bricklayers, more than half of the complaints of shoulder/upper arm, knee/upper leg, neck and back were recurrent. Complaints of the hand and knee recurred most often among the supervisors, with frequencies of 42% (8/19) and 41% (15/37), respectively.

Among the bricklayers with back complaints, 22% (14/64) of the bricklayers reported more problems at the one-year follow-up. Complaints of the upper leg and knee worsened in 50% (4/8) and 26% (11/42) of the bricklayers, respectively.

Also among the supervisors with MSDs was reported that their recurring complaints worsened. This varied according to the body region from 0% (lower arm/wrist, lower leg/ankle, neck) to 40% (6/15) (knee). Complaints of the back that resulted in additional problems at work were reported by 38% (9/24) at follow-up. Among both the bricklayers and the supervisors, approximately one out of the three of the participants with recurrent elbow complaints reported more complaints.

### Occupational physical tasks and activities

Irrespective of the body region involved, bricklayers with MSDs most often report that they perceived the following tasks either caused or aggravated their symptoms: ‘Working with a bent back’ (72%, 128/179), ‘Carrying and lifting’ (64%, 115/179), ‘Working with arms above shoulder height’ (59%, 106/179) and ‘Kneeling and stooping (55%, 98/179). In general, construction supervisors most often pereceived that the following tasks either caused or aggravated their symptoms ‘Carrying and lifting’ (58%, 76/132), ‘Working with a bent back’ (47%, 62/132), ‘Walking across the construction site (44%, 58/132), and ‘Standing’ (42%, 55/132).

In addition to the general differences between occupations, differences were also found for each of the occupational tasks and activities (Table 4). The task ‘Kneeling and stooping’ is perceived among workers in both occupations perceived as the most important factor related to knee complaints (among 63/74 (85%) of the bricklayers and 28/37 (76%) of the supervisors).

**Table 4:** Reported occupational tasks and activities as causes or aggravating factors for musculoskeletal complaints in specific body regions among bricklayers and supervisors participating in a baseline and follow-up survey

**Activity**	**Body region no 1**	**Body region no 2**	**Body region no 3**
**Standing**			
*Bricklayers*	Foot	Lower leg/ankle	Upper leg
*Construction supervisors*	Upper leg	Hip	Back
**Walking across the construction site**			
*Bricklayers*	n.a.	n.a.	n.a.
*Construction supervisors*	Upper leg	Lower leg/ankle	Knee
**Kneeling and stooping**			
*Bricklayers*	Knee	Upper leg	Lower leg/ankle
*Construction supervisors*	Knee	Upper leg	Hip
**Working with a bent back**			
*Bricklayers*	Back	Hip	Upper leg
*Construction supervisors*	Back	Upper leg	Hip
**Carrying and lifting**			
*Bricklayers*	Back	Upper leg	Elbow
*Construction supervisors*	Lower arm/wrist	Back	Upper leg
**Repetitive arm -hand movements**			
*Bricklayers*	Elbow	Lower arm/wrist	Shoulder/upper arm
*Construction supervisors*	Elbow	Lower arm/wrist	Hand
**Working above shoulder height**			
*Bricklayers*	Shoulder/upper arm	Neck	Elbow
*Construction supervisors*	Shoulder/upper arm	Neck	Lower arm/wrist
**Working with vibrating hand tools**			
*Bricklayers*	Elbow	Hand	Shoulder/upper arm
*Construction supervisors*	Hand	Lower arm/wrist	Back
**Climbing ladder/scaffold**			
*Bricklayers*	Upper leg	Lower leg/ankle	Foot
*Construction supervisors*	Upper leg	Knee	Lower leg/ankle
**Driving vehicles**			
*Bricklayers*	Lower leg/ankle	Foot	Hand
*Construction supervisors*	Neck	Back	Hip

*n.a.* not applicable, bricklayers were not asked about the activity ‘walking’.

The top-three body regions for every activity are presented.

Among the workers in both occupations, several participants reported other causes for their MSD. Most of these other causes were not work-related causes, including sports activity (n=12) or neural symptoms after a disc herniation (n=10). None of the bricklayers reported distress as a cause or aggravator of their MSD, whereas seven of the supervisors did.

## Discussion and conclusion

In the present study, we found a high prevalence of MSD among construction workers in two different construction-related occupations, bricklayers and supervisors. The majority of MSDs were perceived as work-related by the bricklayers, whereas this was true for half of the supervisors. Irrespective of occupation, participants with MSDs reported substantial problems during work. After the one year follow-up, over one-third of the bricklayers and supervisors reported recurrent MSDs. About one quarter of this population experienced worsened complaints and experienced additional problems during work.

### Strengths and limitations

Earlier research in the construction field showed that construction workers are at risk for MSDs [21,33] and that MSDs are a determinant of early retirement or disability [5,16]. Based on these previous findings and the high prevalence of MSDs found in the present study, the prevention of MSDs among construction workers is of significant importance. However, to target workplace interventions further knowledge on the nature and degree of MSDs is required. Our study provides, based on a random, adequately sized sample of workers an improved understanding of MSDs in the construction industry [30]. We used a sampling strategy based on occupation and verified the participants’ occupation during the previous year. By doing so, we made certain that the results of the present study are not contaminated by other occupations.

However, some limitations regarding the representativeness of the workers participating in the present study must be noted. First, the construction supervisors who responded were older than the Dutch population supervisors. Second, among the bricklayers age might have affected the prevalence of MSDs, possibly leading to a higher prevalence we found. Third, the follow-up data might be contaminated by the finding that the older workers are more likely to participate in the follow-up. On the other hand, we also verified whether the presence or absence of MSDs at baseline might have affected follow-up response, but we did not find an indication for this. Our follow-up data are therefore not likely to be a contaminated by a selection process due to MSDs in itself.

Our response rate at baseline is similar to the response rates in other surveys [9,16,30]. Despite our effort to increase the response rate by sending reminders and incentives, our baseline response rate of 37% is rather low. Therefore, the sample considered in the present study reflects a portion of the population. Based on the finding that more older supervisors responded, it must be concluded that we the younger population construction supervisors is underrepresented in our present study. Future research should therefore try to reach this population, perhaps by considering other survey modalities or incentives more attractive to them. Nonetheless, our response at follow-up was relatively high (80%).

Regarding the generalisability of our results regarding the factors that cause or aggravate MSDs for supervisors, it must be noted that within the occupation of ‘construction supervisor’, differences exist in the number of physical tasks and activities performed during a regular working day. Some supervisors participate regularly or often in manual tasks (mainly those supervisors working on smaller building projects), whereas other supervisors never do so and are mainly or only involved in managerial tasks. Based on our data we are not able to discriminate between the two types of supervisors. Our findings regarding the perceived causing and aggravating activities fit in with previous results on demands and health effects for those occupations. In a systematic review we found for example that frequent and deep bending, lifting and carrying and working with the arms more than 60^o^ elevated are common for bricklayers [7]. In the present study we found that these demands are perceived as causing or aggravating factors for MSDs. On the other hand, the present study showed that a considerable number of bricklayers perceived kneeling and stooping as a cause or aggravator of their MSD, but from the literature this activity did not appear to be that common. For supervisors little was known about physical demands, but the present study illustrates that these demands should not be to easily neglected.

In the present study, we used a self-reporting measure of (work-related) MSDs. Therefore, our six-month prevalence estimate could be higher than the prevalence of diagnosed work-related MSDs [34,35]. Furthermore, there are likely to be seasonal differences in prevalence. Based on literature, it seems likely that musculoskeletal complaints will more prevalent during the winter than during the summer [36], with probably larger differences for the bricklayers then the supervisors [37]. By asking the workers in December/January about their MSDs during the past six months, we aimed at finding a good estimate of workers who might benefit from preventive actions without too much bias due to including only winter or only summer months.

Furthermore, it could be argued that the interpretation of our results regarding the seriousness of the MSDs is limited because we did not describe a variety of dimensions, such as trouble, pain or disability [38,39]. Instead, we deliberately chose to assess the dimension that is, in our opinion, of highest relevance for occupational health care professionals targeting workplace adjustments, namely the extent of the problems experienced during work due to MSDs.

### Implications

We found that among two distinctly different construction occupations more than half of the workers suffer from MSDs in one or more body region. Among the bricklayers, two-thirds of the population reports two or more complaints. Furthermore, approximately 40% of the workers in both occupations report that that they experience many problems during work due to their MSDs, irrespective of their occupation. This suggests that MSDs should be considered a priority in occupational preventive healthcare, irrespective of the type of occupation.

Based on our results, a job-specific approach should be considered. In the first place, bricklayers report that their MSDs are work-related twice as often (81%) as supervisors. As a consequence, our results indicate that the following activities should be targeted for ergonomic improvement among bricklayers: carrying and lifting, working with a bent back and working above shoulder height. For construction supervisors, the activities ‘standing’ and ‘walking across the construction site’ deserve attention among workers with MSDs.

For workers with MSDs, it should be kept in mind that depending on the type of occupation, a closer look at different occupational activities is warranted. For example, bricklayers with MSDs associated with the knee report that kneeling and stooping are the most important factors causing or aggravating their complaints. In addition, supervisors with knee complaints also report that climbing ladders and scaffolds can cause or aggravate their MSDs. A similar reasoning applies for complaints of the back: in both occupations, preventive actions at the workplace should be primarily directed at reducing the amount of working with a bent back and carrying and lifting. For supervisors, the tasks of ‘standing’ and ‘driving vehicles’ should also be considered as a desired point of action. In summary, workers with MSDs may experience problems or the aggravation of their complaints due to their job-specific occupational activities, depending on the body region involved.

### Further research

The present study provides occupational healthcare professionals with knowledge that is applicable for evidence-based decision making regarding the necessity and importance of ergonomic adjustments in construction-related occupations. However, this report represents only one step toward effective prevention of MSDs. It is important to integrate this knowledge into policies related to occupational healthcare for construction workers, such as periodic surveillance of workers’ health [7]. Thereafter, it is necessary to verify whether such workers’ health surveillance leads to behavioural changes and preventive actions at the workplace [40] and ultimately to a reduction of MSDs and improved work functioning.

## Competing interests

The authors declare that they have no competing interest.

## Authors’ contributions

JB was responsible for data collection, statistical analysis, and drafted the manuscript. All authors conceived and designed the study, read and corrected draft versions of the manuscript and approved the final manuscript. HM, JS and MF-D obtained funding for this study. JS and MF-D were the co-principal investigators. All authors read and approved the final manuscript.

## Authors’ information

Judith K Sluiter and Monique HW Frings-Dresen were the co-principal investigators on this project.

## Pre-publication history

The pre-publication history for this paper can be accessed here:

http://www.biomedcentral.com/1471-2474/13/196/prepub
